# Thoughts and Therapies: Melanoma Brain Metastases

**DOI:** 10.3390/cells15090758

**Published:** 2026-04-23

**Authors:** Chaitanya Sanghadia, Milena Nicosia, Caroline Castelino, Neil Talwar, Safwan Kazmi, Jason Ramirez, Vikas Prabhakar, Matthew Lobato, Albert Nguyen, Tomasz Czerkas, Zachary Rundell, Shaan Bhullar, Hunter Hutchinson, Brandon Lucke-Wold

**Affiliations:** 1 Arizona College of Osteopathic Medicine, Midwestern University, Glendale, AZ 85308, USA; chaitanya.sanghadia@midwestern.edu (C.S.); milena.nicosia@midwestern.edu (M.N.); caroline.castelino@midwestern.edu (C.C.); neil.talwar@midwestern.edu (N.T.); safwan.kazmi@midwestern.edu (S.K.); jason.ramirez1@midwestern.edu (J.R.); vikas.prabhakar@midwestern.edu (V.P.); matthew.lobato@midwestern.edu (M.L.); albert.nguyen1@midwestern.edu (A.N.); tomasz.czerkas@midwestern.edu (T.C.); zach.rundell@midwestern.edu (Z.R.); 2 College of Medicine Phoenix, University of Arizona, Phoenix, AZ 85004, USA; shaanbhullar@arizona.edu; 3 Department of Neurosurgery, University of Florida, Gainesville, FL 32611, USA; hutchinsonhunter@ufl.edu

**Keywords:** melanoma, metastasis, brain, *MAPK*, radiotherapy, targeted therapy

## Abstract

Brain metastases are the third most common metastatic site in melanoma patients, with 40% of melanoma patients developing melanoma brain metastasis (MBM). Symptomology of MBM ranges from headaches, neurological deficits, cognitive changes, and seizures, resulting from MBM embedding in areas of highest blood flow following the breakdown of the blood–brain barrier (BBB) via genetic, cytokine, and molecular processes. The BBB is highly restrictive, making MBM difficult to treat. Challenges in MBM treatment are evident in adverse therapeutic effects, such as neurocognitive decline with whole-brain radiation therapy (WBRT), increased risk of radiation necrosis with stereotactic radiosurgery (SRS), and reduced penetration into the brain, which can lead to drug resistance with prolonged use of *MAPK* inhibitors. This review investigates current and novel treatments against MBM, including radiotherapy, chemotherapy, targeted therapies such as *BRAF*/*MAPK* inhibitors, and immunotherapy.

## 1. Introduction

### 1.1. Melanoma

Melanoma is a cancer of the skin affecting melanocytes. Melanocytes produce melanin, a crucial pigment for photoprotection that gives color to the skin, eyes, and hair [1]. Ultraviolet (UV) light exposure damages melanocyte DNA, mutating genes necessary for its growth and division [2]. Increased exposure to UV radiation, such as from tanning beds and lamps, is a strong contributor to melanoma pathogenesis in affected patients [1].

Melanoma accounts for about 1% of skin cancers but is responsible for the majority of skin cancer-related deaths [2]. Melanoma incidence is affected by age, gender, and socioeconomic factors with the highest incidence in males over the age of 60 [3]. Alarmingly, over the past 30 years, young adults have had the highest melanoma mortality rate [3,4].

### 1.2. Melanoma Brain Metastasis

**Figure 1:**
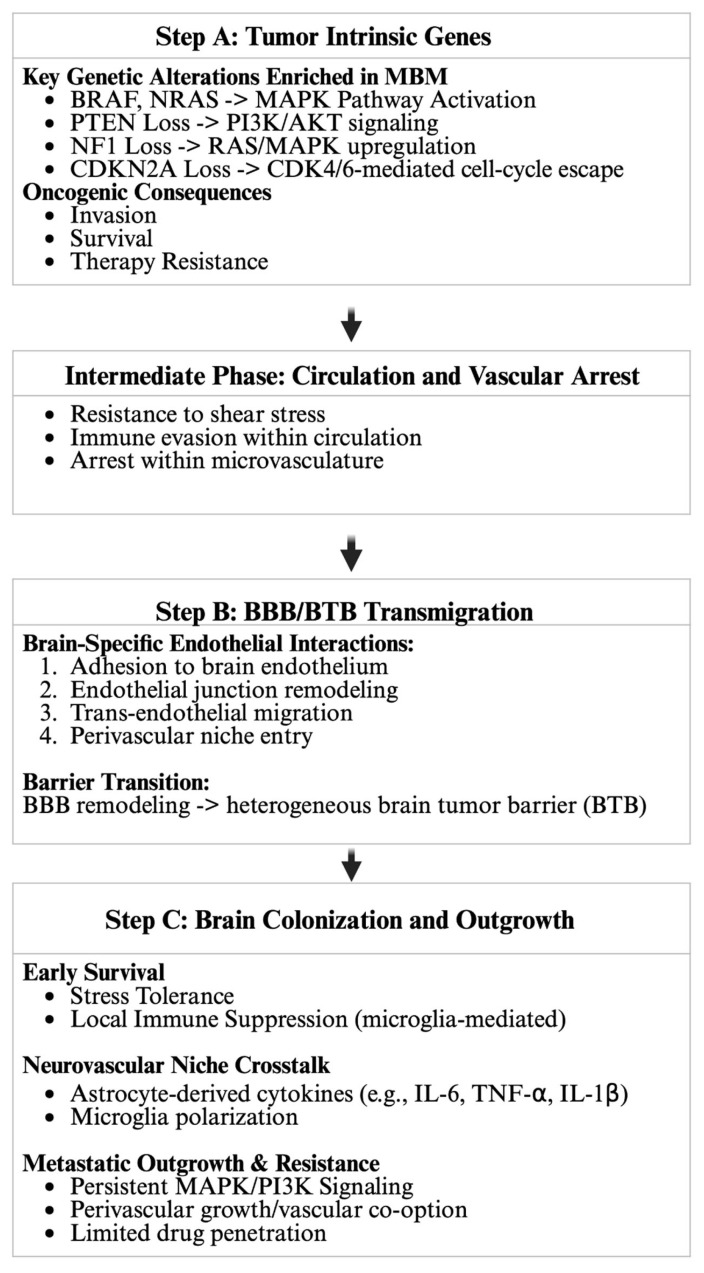
Genetic drivers and multistep mechanisms of melanoma brain metastasis (MBM). Step A highlights tumor-intrinsic alterations enriched in MBM, including activation of *MAPK* signaling (*BRAF* and *NRAS*), dysregulation of the *PI3K/AKT* pathway via PTEN loss, and cell-cycle deregulation associated with *NF1* and *CDKN2A* loss, which collectively promote invasion, survival, and therapeutic resistance. The intermediate circulatory phase depicts hematogenous dissemination, during which circulating tumor cells resist hemodynamic shear stress, evade immune surveillance, and undergo vascular arrest within the microvasculature [22]. Step B illustrates brain-specific endothelial interactions required for blood–brain barrier (BBB) transmigration, including endothelial adhesion, junctional remodeling, trans-endothelial migration, and perivascular niche entry, which result in BBB remodeling and the evolution toward a heterogeneous brain–tumor barrier (BTB) [22,23]. Step C demonstrates early metastatic survival and outgrowth within the neurovascular niche, supported by astrocyte-derived pro-inflammatory cytokines (e.g., IL-6, TNF-α, and IL-1β), microglial polarization, sustained *MAPK/PI3K* signaling, vascular co-option, and limited therapeutic penetration [24,25,26]. Together, these processes define a gene–microenvironment interplay that drives MBM establishment and therapeutic resistance.

Metastatic melanoma commonly involves the GI tract, lung, and bone [3]. Brain metastasis represents one of the most clinically challenging and lethal disease manifestations [3,5,6,7,8,9]. Figure 1 outlines the pathogenesis of melanoma brain metastasis (MBM). MBM occurs through hematogenous dissemination of tumor cells and is mediated by tumor-intrinsic genes that enable survival in circulation [10]. Survival of metastatic melanoma in the circulation depends on interactions with vascular tissue, including the formation of the blood–tumor barrier (BTB) and transmigration across the blood–brain barrier (BBB) [11]. The BBB is maintained by glial cells, specifically astrocytes, which develop a tumor microenvironment by secreting IL-6, TNF-⍺, NF-κB, and IL-1β [6,7,9].

Following dissemination into the brain, the malignant cells tend to embed in areas with the highest blood flow, specifically the cerebral hemispheres (80%), brainstem (5%), and cerebellum (15%), with a historically poor prognosis of 4–6 months before the development of more novel therapies [9,12,13]. Initial presentation of MBM includes headache, neurological impairment, and seizures [13,14]. These symptoms are generated due to risk factors such as male gender, primary disease site in the head or neck, the presence of visceral or nodal metastases, metastases at three or more sites, and elevated LDH levels [13].

MBM is aggressive, therapeutically limited in crossing the BBB, and has numerous complications contributing to its deleterious nature [13]. Cutaneous melanoma is present in more than 50% of stage IV patients. The cancer has the highest brain metastasis rate among other solid tumors [9,13]. Approximately 5% of cutaneous melanomas are diagnosed at stage IV, which has a relapse rate of 29% [15]. Stage IV melanoma is lethal due to the complications of metastasis to the brain, lungs, liver, and bone, with a 28% 5-year overall survival [16]. MBM complications include hemorrhage and focal, global, or functional neurologic symptoms. Focal symptoms are related to paresis, global symptoms are related to intracranial hypertension, and functional symptoms are related to encephalopathy caused by therapeutics or stemming from seizures [13]. The incidence of seizures is higher for MBM, at 19.8%, compared to melanoma patients, at 8.62%, likely due to interactions between tumor cells and the central nervous system [13,14]. MBM has a high hemorrhage risk leading to intracerebral hemorrhage. Basic fibroblast growth factor (bFGF) expression increases the density of immature blood vessels, contributing to MBM-associated hemorrhage neurological sequelae [17,18].

MBM is challenging to treat due to its historical exclusion from clinical trials, despite being estimated to occur in more than 30–75% of patients with melanoma [12,19,20]. Multiple therapeutic options are being developed and utilized for MBM, including combination therapies such as *BRAF + MEK* inhibitors, which have shown high rates of rapid response and improved overall MBM survival. Other therapeutics include immune checkpoint inhibitors (ICIs), such as the anti-CTLA-4/anti-PD-1 combination [13], as well as surgical resection, whole-brain radiotherapy (WBRT), and stereotactic radiosurgery (SRS) [21]. These therapeutic advances directly target the tumor-intrinsic pathways and immune mechanisms highlighted in Figure 1. In this review, we aim to describe the pathogenesis and treatment of MBM due to mutations such as *BRAF, MAPK, PTEN*, and *CDKN2A.*

## 2. Pathogenesis of Melanoma Brain Metastasis

### 2.1. Key Mutations in MBM

MBMs are associated with mutations in key oncogenic pathways, including activating mutations in *BRAF*, *NRAS*, and the mitogen-activated protein kinase (*MAPK*) pathway, as well as loss of tumor suppressors such as phosphatase and tensin homolog (*PTEN*), *NF1*, and *CDKN2A*. These mutations cause dysregulation of the cell cycle, increasing cell survival, invasion, and metastatic competence, thereby supporting survival, proliferation, and adaptive processes associated with integration into the brain’s microenvironment [27,28].

### 2.2. BRAF

*BRAF* is a serine/threonine kinase in the RAF family that is mutated in nearly 50% of melanomas [29]. RAF proteins dimerize to become active, with *RAS* accelerating dimerization, while *ERK* provides negative feedback on the *RAF* dimerization pathway [30,31,32]. The most common *BRAF* mutation is *BRAFV600E*, which causes unregulated downstream *MEK/ERK* activation [33]. The mutation leads to uncontrolled cell proliferation and tumor development. The most common BRAF mutation, V600E, leads to the constitutive activation of the MAPK signaling pathway, promoting cell growth and inhibiting apoptosis, thereby driving malignancy and reactive astrocytes [28,34].

### 2.3. NRAS

*NRAS* is the second most common oncogene in metastatic melanoma [35]. *RAS* proteins are small GTPases implicated in oncogenesis, including *KRAS*, *HRAS*, and *NRAS* [36]. *RAS*-guanine nucleotide exchange factors (GEFs) catalyze GDP-to-GTP exchange, leading *RAS* proteins to bind *RAS*-binding domains, which include *BRAF* [37]. Most *RAS* mutations increase the GTP:GDP-bound *RAS* ratio, thereby markedly increasing basal activation of downstream pathways [38]. In addition to promoting tumor growth via BRAF signaling, *NRAS* mutations mediate immune suppression via PD-L1 upregulation, cGAS-STING suppression, and reduction of antigen presentation [39,40,41]. *RAS* also suppresses PTEN, which is a negative regulator of *MAPKs* [42]. Despite success in targeting *KRAS* for other solid tumors, targeting *NRAS* has had little success, and more research is needed to target this important mediator in melanoma growth [43,44].

### 2.4. MAPKs

*MAPKs* are a family of serine/threonine kinases that act as a convergence point for signal transduction pathways [45]. MAP kinase-ERK kinase (*MEK*) and extracellular regulated MAP kinase (*ERK*) are two *MAPKs*, with mutations present in 3–8% of melanomas [46]. *MEK* phosphorylates *ERK*, which stimulates protein synthesis for growth [47]. Mutations in these proteins are not as common as *BRAF* (a MAP kinase kinase kinase) or *NRAS* (a MAP kinase kinase); targeting *MAPKs* is still actionable because they are downstream effectors of more common mutations [47].

### 2.5. NF1

*NF1* encodes neurofibromin, a GTPase that downregulates *RAS* activation [48]. *NF1* mutations are present in 12–18% of melanomas and occur more frequently in older adults with significant UV exposure [48]. This mutation is also present in 45–95% of desmoplastic melanomas, a painful melanoma subtype that often develops on sun-damaged skin [48]. *NF1* mutations are often the driver in melanomas lacking *BRAF* or *NRAS* mutations, making *NF1* a target for resistant MBM [49].

### 2.6. CDKN2A

*CDKN2A* is a tumor suppressor gene mutated in 60–70% of melanomas [50]. This gene encodes p16 and p14. Canonically, p16 inhibits cyclin-dependent kinases (CDKs), preventing Rb phosphorylation, which arrests the cell cycle at the G1/S phase [51]. Recent evidence, cogently reviewed by Buj & Aird in 2019, has also implicated p16 in a variety of other pathways, including *mTORC1*, *p65*, *ROS*, *PRC*, *TFAM*, *JNK1/3*, and *eEF1A2* [52].

The role of p14 is to prevent ubiquitination and degradation of *p53*, a key tumor suppressor that also arrests the cell cycle at G1/S phase [53]. Mutations in *CDKN2A* are mechanistically distinct from those in *MAPK* pathways, making them a potential alternative target for MBM therapies.

### 2.7. Chromosomal Instability

Another factor that influences MBM development is chromosomal instability. Duplications, deletions, and translocations alter chromosome number and integrity, increasing the probability of aggressive tumors crossing the BBB [17]. Disruption of chromosomal integrity can also cause melanoma cells to adapt to the brain’s isolated environment, helping tumors evade immune detection. Chromosomal instability can also increase MBM drug resistance [17,54].

### 2.8. Blood–Brain Barrier and Blood–Tumor Barrier

A key challenge in MBM is the presence of the restrictive BBB and BTB. The presence of these barriers limits the efficacy of chemotherapeutic agents in the brain, preventing them from achieving a meaningful pharmacodynamic effect on their target of interest [9].

The BBB is a semi-permeable structure that prevents the diffusion of most molecules into the brain parenchyma unless the molecule is lipid-soluble and less than 400–600 Da, or has a BBB-specific receptor or transporter [55]. These parameters prevent most molecules and cells, including tumor cells, from entering the brain [56]. This barrier is composed of endothelial cells with a negatively charged glycocalyx, which repels molecules and astrocytes, which regulate BBB function; pericytes, which enhance communication with growth factors; and cell junction proteins, which promote adhesion [56]. MBM cells initially overcome this barrier to metastasize to the brain by upregulating and anchoring to vascular adhesion proteins such as selectins and integrins [57]. Once anchored, these cells secrete extracellular vesicles which deliver miRNAs that break down the cell membrane, such as miR-181c and cytokines via NF-κB [58,59]. After this initial degradation, MMPs derived from MBM cells help degrade the BBB further while transmigrating via a paracellular route to enter the brain parenchyma [60,61].

When tumor cells metastasize to the brain, elevated VEGF levels stimulate angiogenesis, remodeling the BBB. This disrupts the integrity of the BBB, creating a nuanced vascular unit termed the BTB. The BTB disrupts tight junctions, thickens basement membranes, increases fenestrae, and promotes the formation of abnormal pinocytic vesicles, leading to abnormally leaky blood vessels with non-uniform permeability [54,62,63].

Chemotherapy delivery is limited by BBB penetration; however, successful agents have been shown to have limited therapeutic response [64,65]. Astrocytes promote chemotherapy resistance through sequestration of intracellular calcium and increased gap junction communication [7,66].

## 3. Radiotherapy

### 3.1. Background on Radiotherapy

Radiotherapy plays a central role in treating brain metastases because it can quickly palliate neurologic symptoms, decrease edema-related mass effect, and improve intracranial control [67]. Current practice typically favors focal approaches such as stereotactic radiosurgery (SRS) for limited-volume disease due to strong local control and better cognitive preservation, while whole-brain radiotherapy (WBRT) remains important for patients with extensive intracranial tumor burden, lesions unsuitable for SRS/surgery, or when a comprehensive whole-brain approach is needed [68]. WBRT delivers radiation to the entire brain and targets both macroscopic lesions and microscopic disease, while SRS administers highly conformal radiation limited to the surgical cavity, thereby sparing normal brain tissue [69].

### 3.2. Whole-Brain Radiotherapy

WBRT is most often used as palliative therapy for symptomatic patients with diffuse intracranial metastases who are not candidates for focal therapy, with goals of symptom relief and stabilization of neurologic function [68,69]. Conceptually, WBRT treats visible metastases while also addressing potential microscopic/subclinical disease beyond what is detectable on imaging [68]. However, WBRT has historically been limited by neurocognitive toxicity, and guideline syntheses of randomized evidence support that adding WBRT to focal strategies increases cognitive decline without a consistent overall survival advantage in many settings—driving the modern preference for SRS when feasible. SRS provides comparable survival and local control to WBRT while more effectively preserving neurocognitive function [68,70].

In melanoma brain metastases (MBM), WBRT is now generally reserved for selected palliative scenarios (e.g., extensive intracranial disease not amenable to SRS/surgery or progression despite systemic therapy) [71]. Outcomes with WBRT alone are typically modest; a systematic review of radiotherapy in MBM reported median survival ~3.5 months with WBRT alone versus ~7.5 months with SRS alone (noting strong selection effects and heterogeneity across studies) [71]. More broadly, many MBM patients ultimately die from extracranial progression, so WBRT often cannot meaningfully change long-term survival even when it provides short-term intracranial palliation [71].

When WBRT is necessary, modern practice increasingly incorporates neuroprotection strategies. Major guidelines recommend hippocampal-avoidance WBRT (when feasible) with memantine for appropriate patients. The final phase III results of NRG Oncology CC001 show better preservation of cognition and patient-reported symptoms with hippocampal avoidance plus memantine compared with standard WBRT plus memantine [68,72].

### 3.3. Stereotactic Radiosurgery

SRS, an alternative to WBRT, delivers high-dose radiation to discrete MRI-defined targets with a steep dose falloff, limiting dose to the surrounding brain tissue and reducing the likelihood of global neurocognitive decline compared with WBRT [68,70]. This makes SRS well-suited to MBM, where lesions are typically well visualized on contrast-enhanced MRI and can often be treated focally [71].

Important SRS toxicities include radiation necrosis (RN) and intracranial hemorrhage. RN rates vary by dose, volume, and fractionation; contemporary clinical series continue to document clinically meaningful RN in a subset of patients following SRS [73,74,75]. Hemorrhage is particularly relevant for melanoma metastases, with hemorrhage risk increasing over time post-Gamma Knife resection of MBM [76]. For this reason, it is important to monitor and manage hematologic modulators, including platelets, anticoagulation, and corticosteroids.

Novel preclinical trials have shown the efficacy of Novel Microbeam Radiation Therapy (MRT) on radioresistant murine melanoma and its metastases in mouse models. MRT delivers X-rays at a high rate into micrometer-range high-dose areas, separated by a few hundred micrometers of low-dose regions. The spatial dose separation enables high peak doses while mitigating neurotoxicity. MRT’s reduced neurotoxicity and effectiveness warrant future investigation as an MBM treatment [77].

## 4. Immunotherapy

### 4.1. Monoimmunotherapy for Melanoma Brain Metastases

Immunotherapy harnesses the body’s immune system to target cancer cells, providing a systemic approach that differs from traditional tumor-directed therapies. In MBM, immune checkpoint inhibitors targeting CTLA-4 and PD-1 pathways have emerged as critical therapeutic agents [78,79]. Ipilimumab (anti-CTLA-4) was among the first to demonstrate intracranial efficacy, enhancing T-cell activation and resulting in notable tumor regression in select patients, with an intracranial response rate (ICRR) of approximately 16% and a median survival of approximately 7 months [78]. Pembrolizumab and nivolumab (anti-PD-1 inhibitors) further advance this approach by reversing T-cell exhaustion, achieving an ICRR of 20% to 22%, and prolonging median survival to approximately 18.5 months in asymptomatic MBM patients [79].

Immune-related adverse events (irEAs) have been observed after immunotherapy, with irEAs appearing more frequently after combination therapy as opposed to monotherapy [80,81]. It is hypothesized that irAEs arise from cross-reactivity to shared antigens between tumor and normal cells, leading to T-cell-mediated responses at sites outside the tumor. Alternatively, decreased systemic tolerance to self-antigens induced by immunotherapy could be the source of irAEs [82]. Clinical trials, including CheckMate-204, reported ICRR up to 51% and a two-year survival rate of nearly 72% in asymptomatic patients, representing a substantial advancement over monotherapy [79,83]. This approach has significantly transformed MBM management and outcomes [84]. However, for symptomatic patients, ICRR was only around 22.2%. In these patients, radiation therapy or surgery may be necessary before immunotherapeutic treatment, as steroids may potentially interfere with their efficacy [85,86].

### 4.2. Combination Immunotherapy

Combination therapy using dual checkpoint inhibitors (nivolumab + ipilimumab) has significantly improved outcomes [87]. This combination leverages a “priming and boosting” mechanism: CTLA-4 blockade broadens T-cell activation, while PD-1 blockade sustains effector T-cell function, resulting in higher intracranial response rates (46–57%) and durable disease control, even in the CNS [88]. The American Society of Clinical Oncology, in collaboration with the Society for Neuro-Oncology (SNO) and the Society for Neuro-Oncology (ASTRO), recommends ipilimumab with nivolumab as a standard first-line option for patients with asymptomatic melanoma brain metastases, with monotherapy (nivolumab or pembrolizumab) as an alternative [89].

Immunotherapy is also increasingly combined with stereotactic radiosurgery (SRS). The concurrent use of immune checkpoint inhibitors and SRS leverages radiation-induced tumor antigen release to potentiate systemic immune responses (the abscopal effect), enhancing ICRR and tumor control compared to either therapy alone [90,91]. Emerging data suggest optimal timing, generally within two weeks of SRS, may maximize therapeutic synergy without significantly increasing toxicity [92]. Surgery remains important for selected patients with limited, symptomatic, or surgically accessible lesions.

While combination immunotherapy has been noted to improve the survival rate in a number of MBM patients, it can also come at the cost of acquired resistance mechanisms. Acquired resistance can occur through mutations in the Janus Kinase 1 and 2 enzymes (JAK1/2), which has the effect of reducing expression of PD-L1 and lowering antigen presentation to immune cells. Additionally, mutations of the β2-microglobulin can result in loss of MHC I expression on the cell surface and decreased recognition by immune cells [87].

## 5. Targeted Therapies

### 5.1. MAPK Inhibitors

Targeted therapies against *MAPK* pathway components such as *BRAF* and *MEK* are standard for treating *BRAF* V600 melanoma brain metastases. *BRAF* inhibitors targeting the V600E variant are effective in treating melanoma due to the prevalence of the mutation [93]. Notable FDA-approved *BRAF* inhibitors include dabrafenib, encorafenib, and vemurafenib [94]. The *MEK* inhibitors trametinib and cobimetinib target *MEK1/2* and reduce their downstream targets [93]. These drugs bind to the ATP-binding site of the *BRAF* V600E kinase domain and *MEK*, respectively, preventing downstream signaling [93,94]. ERK inhibitors are in clinical development, with the most advanced ERK1/2 inhibitor ulixertinib currently in phase I and II trials [95]. Combination targeted therapy has shown greater efficacy,

*MEK*/*BRAF* inhibitor combination therapy is superior to monotherapy because it delays resistance and mitigates toxicities specific to *BRAF* inhibitor therapy. A real-world multicenter respective cohort study of 65 patients used three *MEK*/*BRAF* therapies, showing a progression-free survival from the start of therapy of 5.3 months with a median overall survival of 9.5 months [96]. Combination therapy with *MEK* and *BRAF* inhibitors is a viable treatment option for patients dealing with *BRAF*-mutant MBMs [96,97].

Secondary mutations cause therapeutic resistance in *MAPK* inhibitor therapy after 6–9 months. According to a novel study by De et al., machine learning algorithms found drugs that interact with *BRAF* V600E could circumvent limitations of traditional therapies [94]. Additionally, *MAPK* inhibitors increase T cell activity and tumor antigen expression, allowing for adjunct immunotherapy [98]. These inhibitors may be sequenced or combined with immunotherapy to treat BRAF-mutant melanomas, and the optimal strategy is currently under investigation [98,99]. While *RAS* mutations have been historically difficult to target, daraxonrasib is a novel *RAS* inhibitor that may have future applications to MBM [41,100].

### 5.2. Other Targeted Therapies

Targeted therapies for MBM outside the *MAPK* pathway are under investigation for MBM and other tumor types, but have yet to be approved. Direct PTEN inhibitors, such as bisperoxovanadium compounds, vanadyl-hydroxypicolinic acid, and SF1670 are small molecule inhibitors in preclinical development [101,102,103]. Tumor suppressor genes, such as *NF1* and *CDKN2A*, are more difficult to target pharmacologically because they require recovery rather than inhibition of a molecular pathway. Novel drug discovery methods using artificial intelligence (AI) may uncover more therapeutic tools against MBM. Further research is needed to validate the involvement of AI in MBM treatment. Table 1 compares different treatment modalities for MBM. Table 2 lists targetable mutations in MBM, molecules inhibiting these targets, and the clinical status of these molecules.

## 6. Discussion

Melanoma brain metastasis is the third most common brain metastasis, driven by tumor-intrinsic genetic alterations and supportive inflammatory signaling [104,105]. Treatment for MBM has evolved over the years to include radio therapies such as SRS and WBRT [68,69,70,71]. The BBB and cancer-protective properties of astrocytes limit chemotherapy efficacy against MBM despite its use in other cancers [66]. More novel therapies, such as immunotherapy and *MAPK*-targeted therapies, aim to reduce toxicity and improve treatment outcomes [78,79,88].

WBRT has historically been used as a palliative external-beam option for patients with diffuse or symptomatic brain metastases because it treats both visible lesions and potential micrometastatic intracranial disease [73,74,75]. However, in melanoma brain metastases, WBRT is now generally de-emphasized because durable intracranial control is limited and the risk of neurocognitive decline and quality-of-life deterioration is substantial. Current practice guidelines increasingly recommend stereotactic radiosurgery (SRS) (and postoperative cavity SRS, when appropriate) as the preferred radiation strategy for many patients, often including those with multiple metastases, to maximize local control while minimizing cognitive toxicity [89]. WBRT is typically reserved for carefully selected circumstances such as widespread intracranial disease not amenable to SRS, poor prognosis, limited life expectancy, where short-course palliation is the goal, or salvage scenarios. When WBRT is used, cognitive-sparing approaches such as hippocampal avoidance and memantine are recommended when feasible to reduce treatment-related neurocognitive morbidity [69,99].

Another treatment modality targeting cancer cells is immunotherapy that blocks CTLA-4 and PD-1, which are inhibitory receptors that allow cancer cells to evade detection by the immune system. Immune checkpoint inhibitors like ipilimumab (anti-CTLA-4) enhance T-cell activation and proliferation, prolonging them and achieving an intracranial response rate (ICRR) [78]. Both pembrolizumab and nivolumab (anti-PD-1 inhibitors) have shown even greater efficacy [79,88]. Additionally, checkpoint inhibitor combination therapy (nivolumab and ipilimumab) yields significantly improved outcomes, including a higher ICRR and more robust disease control, even in the CNS. The ASCO recommends the combination of ipilimumab and nivolumab as the first-line treatment for patients with asymptomatic melanoma brain metastases, with monotherapy (nivolumab or pembrolizumab) as an alternative [87,89]. Furthermore, the administration of immunotherapy alongside SRS is becoming increasingly common. Concurrent use of immune checkpoint inhibitors and SRS leverages radiation-induced tumor antigen release to enhance systemic immune responses [90,91]. Current data suggest optimal timing, generally within two weeks of SRS, can potentially enhance therapeutic synergy without significantly increasing toxicity [92].

*BRAF* and *MEK* inhibitors treat melanoma by reducing activation of downstream targets [93,94,97]. Combination therapy mitigates *BRAF*-inhibitor-specific toxicities [96,97]. However, these *MAPK* inhibitors can induce resistance through secondary mutations, a risk that may be mitigated by using AI algorithms to select drugs that best target the chosen receptors [99,106].

## 7. Future Directions

Many novel cancer therapeutics are applicable to MBM. Therapies that bypass the BBB involve novel routes of administration, including intranasal drug delivery via the olfactory and trigeminal nerve pathways, as well as regulating the non-uniform permeability of the blood vessels, are especially applicable to MBM. The latter has been explored in which tumor markers have been found against the BTB, including ATP-sensitive K^+^ channels, Ca^2+^-activated K^+^ channels, and ATP-sensitive Ca^2+^ channels, thus allowing the possibility of modulating BTB-associated permeability. Also, components of the tight junctions, such as claudin-5 and angulin-1, have been shown to be promising drug targets that could be delivered against the BBB [107].

Other researchers have explored additional treatment models applicable to MBM, in addition to metastases to other organs. One such strategy leveraged the protective glial machinery to create a glial cancer cell co-culture that identified targetable astrocyte-driven brain metastases associated with melanoma, as well as lung and breast cancer [7]. Another study investigated the use of fluoxetine to inhibit MBM, reporting that it achieved effective growth inhibition across melanoma cell lines without major effects on healthy tissue [108].

These studies demonstrate that mitigating the dire effects of MBM is an ongoing endeavor for researchers. That said, formulating a therapeutic standard for MBM may be on the horizon, but it may only materialize if there is continued interest in clinical research aimed at a therapeutic solution.

## 8. Conclusions

MBM therapy has progressed from WBRT and SRS to newer therapies such as immunotherapy and targeted therapy. Overcoming the BBB and BTB is a key difficulty for delivering therapy. Targeted therapies aim to combat mutations implicated in tumor growth and metastasis, like *BRAF*. Immunotherapies reduce immune-dampening to leverage the immune system for tumor destruction. Researchers are still refining these treatments to improve the standard of care for patients.

## Figures and Tables

**Table 1:** Comparison of treatment modalities for melanoma brain metastases. WBRT = whole-brain radiotherapy; SRS = stereotactic radio surgery; CTLA-4 = cytotoxic T-lymphocyte associated protein 4; PD-1 = programmed cell death protein 1; MBM = melanoma brain metastasis; MAPK = mitogen-activated protein kinases; irAE = immune-related adverse events.

Feature	WBRT	SRS	Immunotherapy (Monotherapy)	Immunotherapy (Combination)	Targeted Therapy (BRAF/MEK)
Treatment Type	Global radiotherapy	Focal radiotherapy	Systemic	Systemic	Systemic
Mechanism	Whole-brain radiation treats macro- and microscopic disease	High-dose conformal radiation to discrete lesions	Immune checkpoint inhibition (CTLA-4 or PD-1)	Dual checkpoint blockade (CTLA-4 + PD-1)	MAPK pathway inhibition (BRAF/MEK)
Typical Indication	Diffuse intracranial disease; palliative	Limited number of lesions; focal control	Asymptomatic MBM	First-line for asymptomatic MBM	BRAF V600–mutant MBM
Intracranial Response Rate	Low	Moderate–high	16–22%	46–57%	Not explicitly stated (rapid responses observed)
Median Overall Survival	3.5 months	7.5 months	7–18.5 months	Improved; 72% 2-year survival (asymptomatic)	~9.5 months
Onset of Effect	Rapid	Rapid	Delayed	Moderate	Rapid
Durability of Response	Limited	Moderate	Moderate	High	Limited (resistance develops)
Key Toxicities	Neurocognitive decline	Radiation necrosis, hemorrhage	Immune-related adverse events	Increased irAEs	Resistance, class-specific toxicities
Special Considerations	Hippocampal avoidance + memantine reduces cognitive decline	Spares normal brain tissue	Steroids may reduce efficacy	Synergistic with SRS; higher efficacy	Resistance after ~6–9 months
Role in Current Practice	Reserved for select/palliative cases	Preferred radiotherapy modality	Alternative if combo not tolerated	Standard first-line (asymptomatic MBM)	Option for BRAF-mutant disease; may be sequenced with immunotherapy

**Table 2:** Status of druggable targets for melanoma brain metastases. “Phase” in status denotes the United States Federal Drug Administration trial phase for any solid tumor type.

Mutation	Drugs	Status	References
BRAF	dabrafenib; encorafenib; vemurafenib	Phase IV	[93,94]
NRAS	daraxonrasib	Phase III	[41,100]
MEK	trametinib; cobimetinib	Phase IV	[93]
ERK	ulixertinib	Phase I/II	[95]
PTEN	bisperoxovanadium compounds; vanadyl-hydroxypicolinic acid; SF1670	Preclinical	[101,102,103]

## Data Availability

No new data were created or analyzed in this study.
